# The Effects of Genetic Variation on H7N9 Avian Influenza Virus Pathogenicity

**DOI:** 10.3390/v12111220

**Published:** 2020-10-28

**Authors:** Szu-Wei Huang, Sheng-Fan Wang

**Affiliations:** 1 Model Development Section, Basic Research Laboratory, Center for Cancer Research, National Cancer Institute, Frederick, MD 21702, USA; szu-wei.huang@nih.gov; 2 Center for Tropical Medicine and Infectious Disease, Kaohsiung Medical University, Kaohsiung 80708, Taiwan; 3 Department of Medical Laboratory Science and Biotechnology, Kaohsiung Medical University, Kaohsiung 80708, Taiwan; 4 Department of Medical Research, Kaohsiung Medical University Hospital, Kaohsiung Medical University, Kaohsiung 80708, Taiwan

**Keywords:** H7N9, avian influenza virus, pathogenicity, HA, NA, virulence, adaption

## Abstract

Since the H7N9 avian influenza virus emerged in China in 2013, there have been five seasonal waves which have shown human infections and caused high fatality rates in infected patients. A multibasic amino acid insertion seen in the HA of current H7N9 viruses occurred through natural evolution and reassortment, and created a high pathogenicity avian influenza (HPAI) virus from the low pathogenicity avian influenza (LPAI) in 2017, and significantly increased pathogenicity in poultry, resulting in widespread HPAI H7N9 in poultry, which along with LPAI H7N9, contributed to the severe fifth seasonal wave in China. H7N9 is a novel reassorted virus from three different subtypes of influenza A viruses (IAVs) which displays a great potential threat to public health and the poultry industry. To date, no sustained human-to-human transmission has been recorded by the WHO. However, the high ability of evolutionary adaptation of H7N9 and lack of pre-existing immunity in humans heightens the pandemic potential. Changes in IAVs proteins can affect the viral transmissibility, receptor binding specificity, pathogenicity, and virulence. The multibasic amino acid insertion, mutations in hemagglutinin, deletion and mutations in neuraminidase, and mutations in PB2 contribute to different virological characteristics. This review summarized the latest research evidence to describe the impacts of viral protein changes in viral adaptation and pathogenicity of H7N9, aiming to provide better insights for developing and enhancing early warning or intervention strategies with the goal of preventing highly pathogenic IAVs circulation in live poultry, and transmission to humans.

## 1. Introduction

Influenza A viruses (IAVs) have been shown to be able to cause pandemic in human populations. Historically, there have been four pandemics in human populations as a result of adaptive evolution of IAVs, Spanish flu in 1918 (caused by H1N1), Asian flu in 1957 (caused by H2N2), Hong Kong flu in 1968 (caused by H3N2), and swine flu in 2009 (caused by H1N1) [1]. Most subtypes of IAVs are classified as avian influenza viruses. It is believed that wild birds are the major natural reservoir of IAVs as most subtypes of IAVs are found to be circulating in wild birds. However, two subtypes of IAVs have been found in bats in recent years, and have led to bats being considered another potential natural reservoir [2]. IAVs display highly diverse subtypes owing to antigenic drift and antigenic shift. The occurrence of antigenic drift could help IAVs accumulate point mutations in the genome, for example, in hemagglutinin (HA) and neuraminidase (NA), and may help IAVs evade host immune responses. The antigenic drift of IAVs is due to a lack of proofreading capability of the viral RNA polymerase [3]. The genetic reassortment of IAVs can generate new viral subtypes through two (or more) IAV subtypes co-infecting in a cell, resulting in an exchange of viral segments. Once the new subtype of IAV is transmitted to a host without pre-existing immunity, the phenomenon is referred to as antigenic shift. Compared to antigenic drift, the occurrence of antigenic shift is less frequent. However, three of the aforementioned pandemics were caused by reassortment IAVs from humans and animals. Therefore, the transmission of reassortment IAVs could cause enormous impacts on public health and economics. 

In March 2013, Gao and colleagues first reported that three patients in China infected with the novel H7N9 virus (one of the novel subtypes of avian influenza viruses, the name of H7N9 is used hereafter instead of avian influenza H7N9 virus) had died due to severe illness, including acute respiratory distress syndrome and multi-organ failure [4]. Since those first H7N9 virus-infected cases reported in 2013, five seasonal waves resulting in human cases occurred in China and caused 135, 320, 226, 117, and 766 infection cases, respectively [5,6]. The fatality rate of the H7N9 virus-infected patients is approximately 40% [5]. The H7N9 virus displays higher internal genetic diversity which has been determined to be due to sequential two-step reassortments within wild birds by phylogenetic analysis. The first reassortment engaged H7N?/H?N9 viruses from Eurasian origin with A/brambling/Beijing/16/2012-like the H9N2 virus from Chinese wild birds. The new H7N9 virus from this first reassortment then circulated in Chinese domestic birds. The second reassortment happened in Chinese domestic birds with incorporation of A/chicken/Jiangsu/ZJ4/2013-like the H9N2 virus and A/Shanghai/1/2013-like H7N9 virus. Two-step reassortments generated several genotypes of the H7N9 virus, including A/Anhui/1/2013 (dominant genotype), A/Shanghai/1/2013, and others [7]. The novel H7N9 virus displayed low pathogenicity (LP) in poultry (such as chickens) in the first four human seasonal waves in China, which allowed the H7N9 virus-infected poultry to transmit the virus to healthy birds without any symptoms, resulting in continuing evolution of LPAI H7N9 viruses. In early 2017, the fifth human seasonal wave happened and H7N9 virus-infected cases significantly increased over the previous human-infecting seasonal waves in China. Notably, the emergence of the highly pathogenic avian influenza (HPAI) H7N9 caused severe outbreaks in poultry and further caused death in most infected poultry [8,9]. The HPAI H7N9 virus quickly spread to eight provinces of China and caused large numbers of human infections. Compared to the LPAI H7N9 virus, the HPAI H7N9 virus has a multibasic amino acid insertion in the HA cleavage site and therefore displays increased viral pathogenicity [8,10]. Moreover, phylogenetic tracing analysis of the fifth human seasonal wave of H7N9 virus infection in 2017 showed that the LPAI H7N9 virus was imported from the Yangtze River Delta Region while the HA cleavage site insertion of multibasic amino acids occurred in the Pearl River Delta Region. The HPAI N7N9 virus reassorted with local H7N9 viruses or the LPAI H9N2 virus, leading to the fifth human seasonal wave in China [10,11]. After the wide spread of the HPAI H7N9 virus in the fifth human seasonal wave, the Chinese government introduced an influenza H5/H7 bivalent vaccine for poultry in September 2017, with major introduction in chickens due to decreased replication efficiency of the H7N9 virus in ducks [12]). The vaccination dramatically decreased the H7N9 virus infection in chickens, and more importantly, there was no new human wave with the H7N9 virus [13]. However, it was found that the HPAI H7N9 virus could circulate in ducks with no or moderate pathogenicity after the massive vaccination of chickens [14,15]. This indicates that the ducks could be a potential carrier of the H7N9 virus and be able to transmit the virus. The re-emergence of the HPAI H7N9 virus has been reported in poultry and lead to one human infection case in 2019, suggesting it may be circulating with a low level in poultry in China and showing antigenic drift [16,17]. Notably, several immune escape mutations which did not previously exist were found in the HA of re-emerged HPAI H7N9 viruses. Therefore, the HPAI H7N9 virus is still a potential threat to cause a pandemic due to the existing selection pressure of vaccine antibodies, possible circulation in non-immunized poultry, and its highly adaptive evolutionary ability. 

In this review, we aim to compile current knowledge and advances on the impacts of genetic substitutions in H7N9 viral proteins, especially focused on the H7N9 viral pathogenicity and host adaption in different species.

## 2. Materials and Methods

In this review, we summarized the latest research on the impacts of viral protein changes in viral adaptation and H7N9 viral pathogenicity. This review collected the information from PubMed, Web of Science, and Google Scholar databases. We used search strings containing a combination of terms including H7N9 influenza virus, adaption, pathogenicity, vaccine, and mutations/substitutions. The search covered publication between 2013 and 2020. Search results were limited to articles published in the English language or with English translation available. Full-text articles were reviewed to access their relevance and quality of the methodology. Articles with the following were excluded: insufficient details in materials and methods; irrelevant or insufficient information related to the review objectives; articles not available in full text for review; articles published in preprint journal. We also searched gray literatures such as WHO websites and announcements, and local ministries of health and centers for disease control and prevention.

## 3. Structure of IAVs

IAVs belongs to the family of Orthomyxoviridae. It carries an envelope and contains negative-sense single-strand RNA (ssRNA) segmented genomes. Eight segmented RNA comprise the RNA genome of IAVs. The viral RNA polymerase consists of three subunits which are encoded by PA, PB1, and PB2. PA, PB1, and PB2 are the largest RNA segments of IAVs. This heterotrimeric viral RNA polymerase is responsible for viral RNA synthesis and replication in infected cells. HA is located on the IAVs surface and can bind with the sialic-acid containing receptor on the cell membrane and enter into cells. NA exists at a small amount on the viral envelope (the HA: NA ratio ranges from 4:1 to 5:1) and serves to release the viral particle and help the virus spread through cleavage of the sialic-acid receptor. Nucleoprotein (NP) binds the viral RNA segments. Matrix protein (M1) is located at the viral inner envelope and could serve as a scaffold to the virion structure. Membrane protein (M2) serves as a proton ion channel for virion internal acidification in the endosome, resulting in viral uncoating. M1 and M2 are encoded by the same viral RNA segment. Non-structural protein 1 (NS1) mediates host antiviral response. Nuclear export protein (NEP) mediates viral RNA export to the cell cytoplasm from the nucleus. NS1 and NEP are encoded by the same viral RNA segment [18].

## 4. Receptor Adaptation

Despite wild aquatic birds being the natural reservoirs for all subtypes of IAVs, there are some IAVs which have been shown to be able to adapt to other birds and even mammals [19,20]. The initial step for IAVs infection is through viral HA attachment to the host cell membrane receptor. However, the host cell receptors for HA binding of IAVs are different between avian and humans. Avian is infected with IAVs through HA binding with the α-2,3-linked sialic acid receptor (avian typed), whereas α-2,6-linked sialic acid receptor (human typed) is bound for human infection. Due to the preference of different sialic acid receptors, cross species transmission of IAVs from avian to human is limited [21]. Since 2013, there have been five human seasonal waves caused by H7N9 virus emergence in China. H7N9 virus infection can be persistently detected in poultry, however there is no report showing that there is sustained human-to-human transmission. Therefore, further adaptions are needed in order to transmit H7N9 virus among humans.

**Figure 1:**
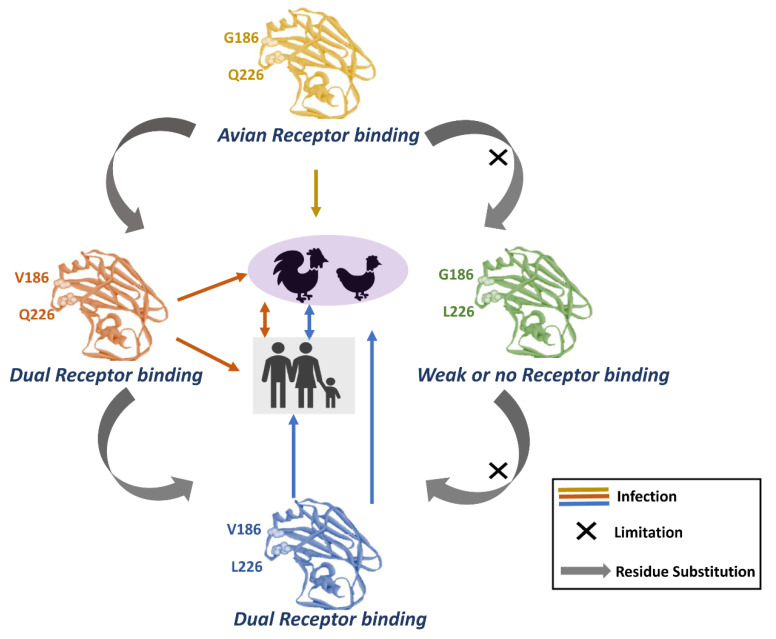
Receptor-binding adaptation of avian H7N9 influenza virus HA. Amino acid substitution on 186 and 226 has effects on change of host adaption.

For efficient transmission of the H7N9 virus to humans, the virus would need to change its HA specificity from the α-2,3-linked sialic acid receptor to the α-2,6-linked sialic acid receptor. Moreover, evidence has shown that the switch of viral HA specificity through amino acid substitutions could increase binding affinity in the upper respiratory tract of humans [22,23]. Before H7N9 virus emergence, several evidences showed that only a few amino acid changes in H5N1 viruses (another subtype of HPAIV which is believed to have potential to cause pandemic) can adapt virus transmission in ferrets [24,25,26]. The occurrence of residue substitutions within the receptor-binding site (RBS) of HA could determine the receptor binding specificity of IAVs [27,28]. Two representative isolates of human-infected H7N9 virus from the early first seasonal wave, A/Shanghai/1/2013 and A/Anhui/1/2013, have been used to study the HA binding specificity. However, A/Anhui/1/2013 is more significantly prevalent in human infections. A/Shanghai/1/2013 has only α-2,3-linked sialic acid receptor binding capacity (avian receptor preference), whereas A/Anhui/1/2013 displays dual binding capacity for α-2,3-linked and α-2,6-linked sialic acid receptors (avian and human typed receptors preference) [29,30,31]. S138A, G186V, T221P, and Q226L (H3 numbering throughout the article) substitutions within the HA RBS of A/Anhui/1/2013 are responsible for changing the binding specificity from avian-specific to dual-specific receptors [29]. A recent study by Xu and colleagues showed that a single G186V mutation is sufficient for the H7N9 virus to acquire α-2,6-linked sialic acid receptor, however the Q226L mutation requires other hydrophobic residues (A138/V186/P221) in order to obtain dual receptor binding affinity. When paired with hydrophilic residues, it can dramatically decrease the binding affinity for both. Therefore, it is believed that adaptive evolution of HA in current H7N9 viruses first occurred at residue G186, and later Q226 substitutions occurred with those three hydrophobic residues [32]. Despite the fact that both Q226L and G186V mutations were found in most human H7N9-infected isolates, the G186V mutation alone is sufficient to change the binding specificity of H7N9 HA from avian typed receptor to dual typed receptors (Figure 1). The prevalence of the H7N9 virus A/Anhui/1/2013 in human infections is possibly due to carrying those mutations in its HA region. An experimental comparison study by Tharakaraman and colleagues showed that the G228S mutation in H3 or H7 HA could change the structural network with S186, T187, and E190, and enhance the binding with both avian and human typed receptors [23]. Despite specific mutations in HA of current H7N9 viruses which could increase binding affinity to human typed receptor; the H7N9 virus did not sustain human-to-human transmission. Thus, it is believed that the H7N9 virus is not yet well adapted in humans. Notably, current evidence has shown that artificially introduced mutations (V186G/K-K193T-G228S or V186N-N224K-G228S) in HA of the H7N9 virus could significantly increase binding affinity from the avian typed to the human typed receptor [33,34]. A comparison of the HA receptor binding pocket of human pandemic IAVs (H1N1, H2N2, and H3N2) and corresponding avian viruses with current human H7N9 viruses showed that the mutations that occurred in 186, 193, 226, and 228 could determine the receptor binding specificity (Table 1). Notably, the H7N9 virus containing G186V and Q226L mutations shows dual receptor binding specificity, however, H7N9 virus acquiring K193T and G228S mutations seems to prefer binding to the human typed receptor. In addition, a recent report by Yu and colleagues found that the V125A mutation in HA (loss of glycosylation site) of the human infected H7N9 virus may associate with the adaptation in humans, as all of the environmental H7N9 isolates (from poultry close to the infected patients) are carrying V125T [17]. T160A mutation in HA can cause loss of glycosylation and it has been found to exist in most H7N9 isolates from infected humans. Moreover, the T160A mutation has been demonstrated to be able to enhance the HA binding affinity to the human typed receptor [22]. The H7N9 virus has been shown to be sporadically transmitted to the human population since 2013 in China, indicating that the highly adaptive evolutionary ability of the H7N9 virus could adapt to the human population. Therefore, constant surveillance and monitoring of the evolution and mutations on the genome of the H7N9 virus (especially the HA gene) is important to develop early prevention, intervention strategies, and to understand the pandemic potential.

## 5. Mutations in Viral Proteins of H7N9 Viruses and the Impacts on Viral Pathogenicity and Transmission

### 5.1. HA

The mutation, insertion, and recombination in the HA cleavage site could determine the pathogenicity of IAVs. During IAVs replication in the cells, the newly produced HA (inactive precursor HA protein) needs to be cleaved into functional HA1 and HA2 by the cellular protease to generate infectious virus particles. It is well known that the number of basic amino acids at the HA cleavage site determines the IAVs pathogenicity and infection specificity of avian cell-types [35,36,37]. However, more than four basic amino acids at the HA cleavage site did not increase the viral pathogenicity in chickens [38,39,40]. LPAIV usually carry one or two nonconsecutive basic amino acids at the HA cleavage site and can be cleaved by cellular trypsin-like proteases. The monobasic amino acid at the HA cleavage site confines the viral replication to respiratory and intestinal epithelial cells. The multibasic amino acid insertion at the HA cleavage site changes the LPAIV to HPAIV by allowing several ubiquitous cellular proteases (furin-like proteases) to process the precursor HA protein, therefore allowing the virus to replicate in cells of multiple tissues and increasing the viral pathogenicity and severity of disease. The HPAI H7N9 virus had multibasic amino acids inserted at the HA cleavage site in the fifth human seasonal wave in China which changed the LPAI H7N9 virus to HPAIV, resulting in more infected poultry death and increased the chance of human exposure and infection from infected poultry [8,10,15]. Evidence has shown that polymerase slippage may facilitate the acquisition of additional basic amino acids in the HA cleavage site [41,42]. A recent study by Gultyaev and colleagues suggests that the conserved stem-loop secondary RNA structure of the HA cleavage site may provide an evolutionary driving force for obtaining multiple basic amino acids [43]. A recent study by Chan and colleagues found that just a multibasic amino acid insertion at the HA cleavage site of H7N9 viruses (from avian) cannot cause disease in mice, however obtaining additional adaptive mutations in HA (A452T), PA (D347G), and PB2 (M483K) after single passage in mice can significantly enhance viral pathogenicity [44]. There is no difference of severity of disease between LPAI and HPAI H7N9 viruses in infected patients, however, avian HPAI H7N9 could be lethal and highly transmissible for mice and ferret after acquisition of the adaptive mutations for mammals [45,46]. Therefore, the mammal adapted HPAI H7N9 virus could increase the chance of virus adaption in humans which poses great threats for public health. Further, to cope with the emerging H7N9 transmission to human, we suggest that prompt infection control and prevention measures should be established, such as gathering the latest infection information, strengthening border quarantine, coordinating medical systems and stockpiles, and managing risk communication.

Other important changes which can affect viral pathogenicity include the glycosylation site in the HA stalk and changes in the H184 or E216 residues in HA. Evidence has shown that changes of the glycosylation site could affect the viral pathogenicity in different IAVs, such as H5N2 [47] and H5N1 [48,49]. In addition, H184 and E216 in HA of IAVs are important for modulating pH of fusion and HA conformation, further affecting the viral pathogenicity [50]. A recent report showed that K64 residue in HA2 of the HPAI H7N9 virus (A/Taiwan/1/2017) is a critical residue for stabilizing HA structure and modulating the pH threshold. Further studies demonstrated that K64 residue in HA2 of the HPAI H7N9 virus can enhance the virulence in mice [51]. Several HA mutations in H7 subtypes of IAVs can affect the HA acid stability, however the HA of the current H7N9 viruses does not carry these mutations [52].

### 5.2. PB2

The major role of PB2 protein in viral replication is to generate 5′ capped RNA fragments through binding to the cap of the host pre-mRNA molecule which is then cleaved by PA for further use as primer for viral transcription [53,54]. The PB2 protein also shows the ability to mediate type I interferon (IFN) expression [55]. After enhancing the HA binding affinity of the H7N9 virus to human typed sialic acid receptor through adaptive mutations, viral PB2 plays a vital role in adapting virus replication in the mammalian cells. The occurrence of adaptive mutations in viral PB2 could help the virus with passing through the species barrier and replicating efficiently in mammalian cells, further enhancing the viral pathogenicity [56,57,58,59]. The I292V, T271A, A588V, Q591K, E627K, and D701N mutations in PB2 have been seen in current H7N9 viruses adapted to replicate in mammalian cells [11,45,60,61,62,63,64,65]. One of those mutations, the E627K mutation in PB2 has been shown to play a pivotal role in adaptive replication of IAVs in mammalian cells [66], such as H5N1 [67] and others [68,69,70]. The E627K and D701N mutations could be obtained by the H7N9 virus while replicating in mammals. The E627K mutation has been shown to enhance viral polymerase activity in mammalian cells [71,72]. However, obtaining the E627K mutation in PB2 during the viral replication in mammals is dependent on two factors, viral PA and host ANP32A proteins. If viruses carry low polymerase activity of PA or lack ANP32A protein in the cells, the H7N9 virus will lose the ability to acquire the E627K mutation in PB2 while replicating in mammals. ANP32A is a host protein which may interact with viral PB2 [73]. ANP32A in mammalian cells may restrict IAVs polymerase activity, however the E627K mutation compensates the restriction [74,75]. Given the importance of ANP32A in IAVs adaption, Domingues and colleagues found that the splicing profiles of ANP32A in avian and mammal species are associated with viral PB2 adaptive evolution. Moreover, a mathematical model has been created to predict the IAVs adaption through viral PB2 signatures [76]. The molecular mechanism of PB2 and ANP32A interaction and its effect on viral replication in avian and mammals still needs further investigation.

It is well known that the E627K and D701N mutations in PB2 of H7N9 virus could dramatically enhance the viral pathogenicity in mammals [60,61,62]. Intriguingly, Yamayoshi and colleagues found that almost all current H7N9 viruses in infected humans contained Q591K, E627K, and D701N mutations in the PB2 region [62]. The H7N9 virus carrying the E627K mutation in PB2 increased the viral polymerase activity in mammalian cells and enhanced the severity of disease in infected mice. If the H7N9 virus lacks the E627K mutation in PB2, the viral polymerase activity and virulence can be compensated for through acquisition of other PB2 mutations (Q591N, T271A, and D701N) in mammalian cells [60,62]. Moreover, Zhu and colleagues found the H7N9 virus containing E627K/D701N dual mutations in PB2 can cause higher viral polymerase activity and more severe disease than the virus carrying a single mutation [77]. Additionally, numerous studies have shown that different single or combinations of mutations in viral polymerase, such as K526R, A588V, or K482R/A588V mutations in PB2 protein with the K497R mutation in PA protein, could contribute to increase polymerase activity and viral pathogenicity in humans and other mammals [63,78,79]. Importantly, the proportion of A588V mutation significantly increased in the fifth seasonal wave in China [80]. The A588V mutation in PB2 protein could be a marker for identifying the HPAI H7N9 virus. However, the in vivo biological features of mutations in the viral protein related to its pathogenicity in mammalian cells need further investigation.

### 5.3. NA

The HA of IAVs is responsible for binding with the sialic acid receptor on the host cell membrane, whereas the NA serves as a sialidase which can cleave sialic acid from glycans and release the IAVs particles [81]. The HA and NA functional balance is an important factor in determining the viral transmissibility and replication. The length in the NA stalk of different IAVs subtypes can significantly affect the viral adaption to different species and its characteristics, especially the HA and NA functional balance [82]. Decreased length of the NA stalk region has been found in other IAVs. Early studies showed that an 18-amino-acid deletion in the NA stalk in IAVs X-7(F1) and lack of a NA stalk in A/WSN/33 (H1N1) resulted in significantly less NA activity and inefficiently eluted virus from erythrocytes [83,84]. Several evidences have found that the length of the NA stalk is related to virulence and the range of host specificity. A 19-amino-acid deletion in the NA stalk of H5N1 enhanced the virulence in mice and it has been speculated that it could contribute to virulence in humans [85,86]. A 21-amino-acid deletion in the NA stalk of H9N2 enhanced the virulence in quail, chickens, and mice [87]. Another 3-amino-acid deletion was found in the NA stalk of H9N2 which increased NA activity, virus elution from erythrocytes, and virulence in chickens and mice [88].

A 5-amino-acid deletion (position 69–73) was found in the NA stalk of current H7N9 viruses, and it was further speculated that it could affect the virus adaption and virulence [89]. However, Bi and colleagues generated different lengths of the NA stalk of H7N9 virus and found that the 5-amino-acid deletion (position 69–73, which naturally exists in the H7N9 virus) did not affect the NA activity, virulence, and viral pathogenicity in mice, whereas the longer deletions (position 49–68, 54–72, and 54–73 which have shown enhanced virulence in H5N1 in previous studies [85,86]) significantly increased the viral pathogenicity in mice [90]. A current study by Park and colleagues applied a 9-amino-acid deletion (position 57–65) or N63T mutation (deglycosylated mutation) in the NA of a human-infecting avian influenza H7N9 virus and showed enhanced viral pathogenicity in mice, indicating that both the length of NA stalk and the glycosylation in NA are important for viral pathogenicity [91]. The molecular mechanisms of how different lengths of NA stalk and the HA-NA functional balance in IAV subtypes affect the host specificity and pathogenicity should be further investigated. Despite the natural occurrence of a deletion in the NA stalk of the H7N9 virus, it did not show any impact on viral pathogenicity, however longer deletions could happen due to antigenic drift and shift. Therefore, sustained surveillance should be considered of any further genetic change in the NA stalk region of the H7N9 virus and the impact on the viral pathogenicity in different hosts should be further investigated.

### 5.4. PB1-F2

PB1-F2 protein is encoded by a +1 alternate open reading frame of PB1 protein and displayed as a small protein (molecular weight of 10.5 kDa with 87 amino acids of A/Puerto Rico/8/34, also called PR8), which was discovered as an abundant epitope recognized by CD8^+^ T cells of PR8 H1N1 virus [92]. The PB1-F2 protein of IAVs is a virulence factor which is responsible for preventing host innate antiviral responses and is only expressed in IAVs infected cells. The PB1-F2 protein localizes to the inner mitochondrial membrane through interactions with MAVS by its C-terminal region, resulting in decreased mitochondrial membrane potential and enhanced cell apoptosis [93]. Additionally, PB1-F2 protein increases the susceptibility of secondary bacterial infection in infected hosts [94,95]. The length, variation, and absence of PB1-F2 in different IAVs subtypes are correlated to the viral virulence. The virulence contributed by the PB1-F2 protein is host and cell type specific [96,97,98,99]. Conenello and colleagues found that a N66S mutation in the PB1-F2 protein exists in both the Hong Kong 1997 H5N1 virus and 1918 pandemic A/Brevig Mission/18 virus, and further demonstrated the N66S mutation can significantly enhance the viral pathogenicity in mice [100].

**Figure 2:**
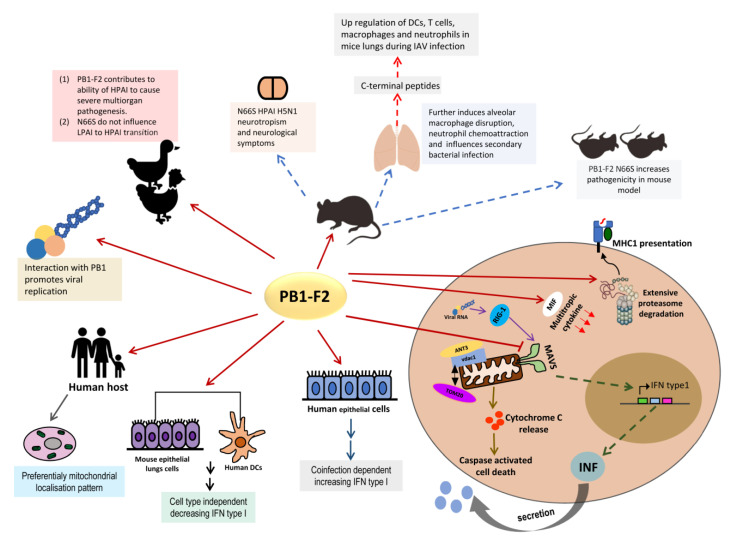
The multiple roles of PB1-F2 participate in regulation of influenza A virus induced pathogenesis.

Moreover, evidence has shown that the N66S mutation in the PB1-F2 protein can enhance the immunopathogenesis in infected mice through suppression of the RIG-1/MAVS signaling pathway, delayed activation of interferon-stimulated gene expression, and decreased mitochondrial membrane potential [93,101,102]. However, the N66S mutation did not exist in the PB1-F2 protein of the current HPAI H7N9 virus. It is believed that the PB1-F2 protein of the HPAI H7N9 virus has evolved different virological and biological features to affect the immunopathogenesis through strong selection pressure. Recent evidence by Cheung and colleagues found a novel mechanism for inhibition of the type I IFN production by the PB1-F2 protein of HPAI H7N9 virus, and its effect on the proinflammatory cytokine storm. The PB1-F2 protein of the HPAI H7N9 virus binds to MAVS and inhibits the aggregation of MAVS, thus reducing MAVS polyubiquitunation. This blocks the activation of antiviral signaling cascades and type I IFN expression [103]. Moreover, Cheung and colleagues also showed that the PB1-F2 protein of the HPAI H7N9 virus suppressed IL-1β secretion through prevention of the RNA-induced NLRP3 inflammasome activation. More importantly, extracellular PB1-F2 peptide induced AIM2 inflammasome or NLRP3 inflammasome was not affected by the intracellular HPAI H7N9 PB1-F2 protein [104]. In generally, the suppression of type I IFN expression, PB1-F2 peptide induced NLRP3 inflammasome maturation, and excessive IL-1β production contribute to severe immunopathogenesis. Figure 2 shows the PB1-F2 protein involved in regulating multiple steps of viral pathogenicity.

### 5.5. PA-X

PA-X is a fusion protein discovered in 2012. The PA-X protein consists of the N-terminal endonuclease domain (191 amino acids) of the PA protein and a unique C-terminal domain obtained from a +1 ribosomal frameshift in the PA gene. The PA-X protein plays a pivotal role in decreasing host protein synthesis in infected cells, a phenomenon also called host shutoff. The host shutoff activity of the PA-X protein can help the virus escape host innate and adaptive immune responses and enhance viral pathogenicity [105]. Evidence has shown that the amino acids in both N- (positions 51–74 and 85–186 amino acids) and C- (positions 192–206 and 232–252) terminals of PA-X are important for global host shutoff activity [106,107,108,109,110]. However, the impacts of host shutoff activity of PA-X protein are strain and host specific, for example, loss of PA-X protein in H1N1 (1918 pdm and swine) and H5N1 can increase virulence in mice, whereas in H9N2 (avian) and H1N2 (swine), loss of PA-X can decrease virulence in mice and pigs, respectively [111]. NS1 protein is another factor which contributes to early nuclear phase host shutoff (the host shut off activity of PA-X protein is involved in late cytoplasmic phase). It is believed that the balance of NS1 and PA-X proteins contributes to global host proteins synthesis, can determine the viral replication and pathogenicity [112,113], and could explain the difference of virulence among IAVs strains. Compared with H5N1, H5N6, and H9N2, the amino acid positions 37, 61, 63, 96, 100, and 101 in the N-terminal of the current H7N9 viruses PA-X gene display higher variation, and notably, positions 61, 63, and 100 are important for the host shutoff activity.

At the C-terminal of the current H7N9 viruses PA-X gene, the amino acid positions 193, 194, 195, 199, 228, and 248 have higher variation, with the amino acid positions 195 and 199, contributing to host shutoff activity [111]. A recent reverse genetic study by Sun and colleagues found the R195K, K206R, and P210L mutations in the PA-X protein of the H9N2 virus enhanced the viral replication and pathogenicity in mice and ferrets. Further focus on the R195K mutation in current H7N9 viruses PA-X protein showed that it can enhance the viral replication and transmission in ferrets. However, the R195K mutation in the PA-X protein of the H9N2 virus did not affect the virulence in infected chickens, indicating the impacts of the mutation could be mammal specific [114]. It will be interesting to investigate the genetic variation of the PA-X gene between HPAI and LPAI H7N9 viruses, and the impacts on viral replication, transmission, host specificity, and pathogenicity. Further study is required to understand the underlying mechanisms of the PA-X protein on host shutoff, transmission, and pathogenicity in different IAV strains, especially on different hosts. Due to the important roles of PA-X in viral replication, virulence, and host immune responses, it could be an ideal target for developing and designing antiviral drugs and vaccines.

### 5.6. Other Viral Proteins, Factors, and New Mechanisms

The NS1 protein of IAVs is an RNA-binding protein and plays a vital role in regulating antiviral host innate immune responses, which include preventing cytoplasmic viral RNA sensor activation [115], preventing host mRNA synthesis, processing, and trafficking [116,117,118], and targeting host proteins involved in the interferon signaling pathway [119,120,121]. Wang and colleagues found that I178 and S212 in the NS1 protein of current H7N9 viruses are important to stabilize the NS1 protein and to restrict host innate immune responses, therefore contributing to the higher virulence of H7N9 virus in humans [122]. Moreover, the P42S mutation in the NS1 protein of current H7N9 viruses has previously been found to be able to increase virulence of H5N1 [123], however the role of the P42S mutation in the NS1 protein on current H7N9 viral pathogenicity needs further investigation. 

The functions of NP are binding and protecting viral RNA, forming viral ribonucleoprotein (vRNP) for viral mRNA synthesis, and helping in the nuclear import event. A recent reverse genetic comparison study by Ma and colleagues found A286 and T437 in NP of current H7N9 viruses can enhance virulence in mice, whereas V286 and M437 substitutions eliminated the virulence of current H7N9 viruses [124]. Notably, A286 and T437 have been found to exist in the NP region of current HPAI H7N9 viruses. Therefore, the NP of the H7N9 virus could be a potential target for antiviral drugs or vaccines. The M1 protein is important for nuclear import, and export of vRNP. In virion, the M1 protein plays a vital role in the structure of viral particles. There are two mutations in the M1 protein (N30D and T215A) which have been found in the current H7N9 viruses. These mutations have been shown to be able to increase H5N1 pathogenicity [125]. Additionally, the E156D mutation in the M1 protein of current H7N9 viruses has been shown to be important for transmission in mammals [65]. Finally, Zhao and colleagues found several adaptive mutations which can enhance virulence of the H7N9 virus through multiple passages of A/Shanghai/2/2013 in mice, which included PA, HA, NP, NA, and M1 proteins [126]. This supports the idea that enhanced virulence through adaptive evolution of the H7N9 virus in humans is of global concern as a potential pandemic threat. Table 2 summarizes the amino acid substitutions in viral proteins of the H7N9 virus which can affect the viral pathogenicity.

In addition, age was recently found to be correlated with H7N9 pathogenicity. A study used nonhuman primates as the model to address the correlation between age and H7N9 pathogenicity and indicated that old aged animals (defined as age 20–26 years) experienced more severe symptoms than infection of young animals (defined as age 2–3 years) [127]. The other study utilized whole transcriptome sequencing (RNA-chip and RNA-seq) and other biomolecular methods to analyze and verify remarkable changes of host cells during LPAI and HPAI H7N9 viruses infection, indicating that up-regulated programmed cell death 1 (PD-1) pathways were found to be changed significantly with several highly correlated non-coding RNAs, contributing to high pathogenicity [128]. Exosomes are known to play an important role in viral infection. Studies have shown that exosomes often carry viral RNA and proteins. Wu et al. recently found that A/Guangdong/GZ8H002/2017 (H7N9) was more pathogenic than A/Zhejiang/DTID-ZJU01/2013 (H7N9) and resulted in the death of mice, which might be due to induction of more exosome secretion [129].

## 6. Conclusions and Perspectives

Since the emergence of the H7N9 virus in China in 2013, it has been responsible for five human seasonal waves, with a fatality rate of around 40% in the H7N9 virus-infected patients. The LPAI H7N9 virus contributed to the first four human seasonal waves from 2013 to 2016; however, the HPAI H7N9 virus emerged during the fifth human seasonal wave in 2016/2017 and with the LPAI H7N9 virus, caused significant increases in human cases due to the HPAI H7N9 virus being more widespread in poultry. The HPAI H7N9 virus also caused more infected poultry death. Evidence has shown that some amino acid substitutions, such as G186V, Q226L, and G228S, in the HA region of current H7N9 viruses are sufficient to change the binding specificity from avian typed receptor (α-2,3-linked sialic acid receptor) to human typed receptor (α-2,6-linked sialic acid receptor) or dual receptors. Moreover, evidence showed that the artificial introduction of site mutations could significantly increase binding affinity from avian typed to human typed receptor, such as V186G/K-K193T-G228S and V186N-N224K-G228S. Though several mutations in HA have been shown to be important for affecting the H7N9 virus binding specificity, antigenic drift could occur at any site within the HA region and then affect the binding specificity. Therefore, a comprehensive study introducing different mutations (single or combination) in HA of the H7N9 virus could provide better insight into their effect on binding specificity. Comparing this with current known adaptive mutations in HA of the H7N9 virus through regular monitoring and enhanced active surveillance could help to control H7N9 virus transmission in poultry and to develop early prevention, intervention strategies, and to understand the pandemic potential.

H7N9 viral pathogenicity could be affected by several viral proteins, such as HA, NA, PB2, NP, M1, NS1 and two accessory proteins PB1-F2 and PA-X. The multibasic amino acid insertion in the HA cleavage site of the H7N9 virus emerged in 2017 and changed the LPAI H7N9 virus to the HPAI H7N9 virus. Despite the fact that HPAI H7N9 virus infection can cause more poultry death than the LPAI H7N9 virus, the fatality rate of humans infected with the HPAI H7N9 virus did not show significant differences. Therefore, it seems the the HPAI H7N9 virus has not yet fully adapted to humans. However, due to the highly transmissible and pathogenic nature of the HPAI H7N9 virus in infected poultry, the wide spreading of the HPAI H7N9 virus in poultry can cause significant increases in human infections. A range of amino-acid deletions in the NA stalk of IAVs can affect the adaption and virulence of the virus. However, a naturally occurring 5-amino-acid deletion in the NA stalk of current H7N9 viruses did not show changes in virulence. It is believed that the H7N9 virus has evolved to achieve the functional balance between HA and NA. Nonetheless, longer amino-acid deletions (19 and 20 amino-acid deletions) inserted artificially into the NA stalk region of the H7N9 virus significantly enhanced the viral pathogenicity. The E627K mutation in PB2 of current HPAI H7N9 viruses can enhance the virulence in mammals through increased viral polymerase activity in most subtypes of HPAIV. Other than the E627K mutation in PB2, introducing multiple mutations also enhanced the viral polymerase activity and virulence. The PB1-F2 protein has been shown to be important in modulating host immune responses. The PA-X protein plays a vital role on host shut off and the R195K mutation can affect virulence and transmission.

The antigenic drift and shift of the H7N9 virus provides a fast mode of adaption to difference species. The mutations in viral proteins determine the viral pathogenicity in infected hosts. Future study is needed to understand the effect of mutations on the viral glycosylation site in HA and NA of the H7N9 virus as protein glycosylation can affect the viral pathogenicity, adaption, and antibody neutralization. Moreover, further investigation into the effect of immune escape mutations in HA on vaccine efficacy is needed due to the recent re-emergence of the HPAI H7N9 virus. A rising concern is two accessory proteins, PB1-F2 and PA-X, which display important roles in the pathogenicity of virus and host immune responses. More reverse genetic study is important to understand the underlying mechanisms of PB1-F2 and PA-X on virulence, transmission, and pathogenicity in different host, especially the differences between HPAI and LPAI H7N9 viruses. Comprehensive active surveillance and identification of mutations associated with high viral pathogenicity will provide better insights on developing early intervention strategies and preventing highly pathogenic IAVs circulating in live poultry, and moreover transmitting to humans. Besides this, the introduction of a vaccine to other poultry (such as ducks) is urgently needed in order to prevent the occurrence of adaptive mutations to humans.

## Figures and Tables

**Table 1:** Amino acid variation in the receptor binding pocket of influenza HAs.

	Amino Acid							Specificity	
Virus Strain	186	190	193	224	225	226	228	α-2,3	α-2,6
Avian H1N1	P	E	S/T	R	G	Q	G	+	−
Avian H2N2	N	E	N	N	G	Q	G	+	−
Avian H3N2	G/V/S	E	N	R	G	Q	G	+	−
H5N1	N	E	K	N	G	Q	G	+	−
H7N9	V *	E	K ^#^	N	G	L *	G ^#^	+	+
Human H3N2	G/V/S	E	T	R	G	L	S	−	+
Human H2N2	N	E	S	N	G	L	S	−	+
Human H1N1	P/S	D	T/A	R	D	Q	G	−	+

Grey indicates amino acids involved in either human or avian typed receptor specificity, whereas blue indicates amino-acid positions that are mutated to the amino acids found in human viruses, correlating with receptor adaption. * G186V and Q226L mutations related to dual receptors binding specificity. ^#^ K193T and G228S mutations related to α-2,6-linked sialic acid receptor.

**Table 2:** Major pathogenicity determinants of the H7N9 virus.

Protein	Main Functions	Mutation	Effect	Host	Ref.
HA	Surface protein, binding to sialic acid receptor of host cell membrane and fusion into the cell, major antigen, related host specificity	Multibasic amino acid insertion at the HA cleavage site	Increase pathogenicity	Chickens	[9,10,15]
		A452T in HA/D347G in PA/M483K in PB2	Increase pathogenicity	Mice	[44]
		E64K in HA2	Increase pathogenicity	Mice	[51]
PB2	Binding to 5′ capped cellular mRNA, regulate the IFN expression	E627K	Increase pathogenicity	Mice	[60,61,62,77]
		D701N	Increase pathogenicity	Mice	[62,77]
		T271A/Q591N/D701N	Increase pathogenicity	Mice	[62]
		K526R	Enhance replication	Mice	[78]
		A588V	Increase pathogenicity	Mice	[63]
		K482R/A588V in PB2 and K497R in PA	Increase pathogenicity	Mice	[79]
NA	Surface protein, a sialidase which can cleave sialic acid from glycans, release the IAVs particles	5-amino-acid deletion in stalk Longer amino-acid deletion in stalk	No effect Increase pathogenicity	Mice	[90]
		N63T	Increase pathogenicity	Mice	[91]
PA-X	Modulation of host shutoff and immune responses	R195K	Increase pathogenicity	Mice, ferrets	[114]
NS1	Regulation of antiviral host innate immune responses and host gene expression, inhibition of cell mRNA translation	V178I and P212S	Increase pathogenicity	Mice	[122]
NP	Binding and protecting viral RNA, regulation of nuclear import event, forms vRNP for viral mRNA synthesis	V286A and M437T	Increase pathogenicity	Mice	[124]
		P42S *	Increase pathogenicity ^#^	Mice	[123]
M1	Contributing nuclear import and export of vRNP, related to viral assembly, budding, and structure of virion	N30D and T215A *	Increase pathogenicity ^#^	Mice	[125]
PB1-F2 ^¶^	Induction of cell apoptosis, suppression of type I IFN expression, enhancement of secondary bacterial infection, suppresses RNA-induced NLRP3 inflammasome activation, and excessive IL-1β production	N66S ^#^	Increase pathogenicity	Mice	[93,94,95,104]
Other factors/mechanisms	Elder aged in animal	NA	Increase pathogenicity	Nonhuman primates	[127]
	Up-regulation of PD-1/PD-Ls pathway-related molecules	NA	Increase pathogenicity	Human	[128]
	Extrapulmonary tissue infection occurs via the exosome pathway	NA	Increase pathogenicity	Mice	[129]

^¶^ There is no study to show the relationship of mutations in the H7N9 PB1-F2 protein and viral pathogenicity. * Mutations frequently found to exist in the H7N9 virus. ^#^ Mutations have shown increased viral pathogenicity in the H5N1 virus, but no related study explores those in the H7N9 virus.
